# Patterns of comorbidity and multimorbidity among middle-aged and elderly women in peri-urban Tanzania

**DOI:** 10.1177/26335565221076254

**Published:** 2022-02-22

**Authors:** Laura-Marie Stieglitz, Till Bärnighausen, Germana H. Leyna, Patrick Kazonda, Japhet Killewo, Julia K. Rohr, Stefan Kohler

**Affiliations:** 1 Heidelberg Institute of Global Health, 9144 Heidelberg University, Heidelberg, Germany; 2 Department of Epidemiology and Biostatistics, 92976 Muhimbili University of Health and Allied Sciences, Dar es Salaam, Tanzania; 3 Dar es Salaam Urban Cohort Study, Dar es Salaam, Tanzania; 4 Harvard Center for Population and Development Studies, 1812 Harvard University, Cambridge, MA, USA

**Keywords:** chronic morbidity, infectious diseases, mental health, multiple chronic conditions, older women, physical health, Sub-Saharan Africa, urbanicity

## Abstract

**Background:**

Multimorbidity poses an increasing challenge to health care systems in Sub-Saharan Africa. We studied the extent of multimorbidity and patterns of comorbidity among women aged 40 years or older in a peri-urban area of Dar es Salaam, Tanzania.

**Methods:**

We assessed 15 chronic conditions in 1528 women who participated in a cross-sectional survey that was conducted within the Dar es Salaam Urban Cohort Study (DUCS) from June 2017 to July 2018. Diagnoses of chronic conditions were based on body measurements, weight, blood testing, screening instruments, and self-report.

**Results:**

The five most prevalent chronic conditions and most common comorbidities were hypertension (49.8%, 95% CI 47.2 to 52.3), obesity (39.9%, 95% CI 37.3 to 42.4), anemia (36.9%, 95% CI 33.3 to 40.5), signs of depression (32.5%, 95% CI 30.2 to 34.9), and diabetes (30.9%, 95% CI 27.6 to 34.2). The estimated prevalence of multimorbidity (2+ chronic conditions) was 73.8% (95% CI 71.2 to 76.3). Women aged 70 years or older were 4.1 (95% CI 1.5 to 10.9) times mores likely to be affected by multimorbidity and had 0.7 (95% CI 0.3 to 1.2) more chronic conditions than women aged 40 to 44 years. Worse childhood health, being widowed, not working, and higher food insecurity in the household were also associated with a higher multimorbidity risk and level.

**Conclusion:**

A high prevalence of multimorbidity in the general population of middle-aged and elderly women suggests substantial need for multimorbidity care in Tanzania. Comorbidity patterns can guide multimorbidity screening and help identify health care and prevention needs.

## Introduction

More and more people in Tanzania reach a high age and live in urban areas.^1,2^ Both can contribute to an increase of multimorbidity in the population. Multimorbidity has been associated with age^3,4^ as people who live longer accumulate chronic diseases and conditions over their lifetime. Living in an urban area has been associated with a growing burden of chronic non-communicable diseases including mental health conditions due to a less healthy diet, a sedentary lifestyle, higher substance abuse, or chronic inflammation.^5–8^

Multimorbidity in Tanzania, as in other Sub-Saharan African countries, is likely to include combinations of infectious and non-communicable chronic conditions as the scale-up of antiretroviral therapy of HIV infection has contributed to improved life expectancy of people living with HIV.^9,10^ Understanding the prevalence of multimorbidity, its composition, and its expected rise—in the general population as well as in special populations—can help prepare health care systems for the financial and structural challenges related to the care of multiple chronic conditions. In previous studies, multimorbidity has been associated with greater mortality and higher odds of hospitalization.^11–17^ In consequence, multimorbidity can lead to a higher utilization of health care and higher costs for the health care system.^15–17^

Women have been considered more likely to be affected by multimorbidity than men.^18–21^ Older women in Sub-Saharan Africa were found to have a higher risk to be affected by non-communicable diseases and depression, more severe functional disabilities, and lower wellbeing than older men.^22^ In addition, women in Sub-Saharan Africa were found more likely to be infected with HIV than men.^23^ At the same time, an estimated 61.3% of women in Sub-Saharan Africa face barriers to accessing health care, often due to financial constraints and large distances to health care facilities.^24^

Few existing studies focused on chronic conditions and multimorbidity in older women in Sub-Saharan African countries^22^ despite their growing number and vulnerability to multimorbidity. The study at hand investigated the patterns of comorbidity and the extent of multimorbidity in middle-aged and elderly women, who lived in a peri-urban area of Dar es Salaam in Tanzania.

## Methods

### Study setting

The study was conducted in the Ukonga ward of Dar es Salaam in Tanzania. It is part of the larger “Health and Aging in Africa: Longitudinal Studies in three INDEPTH Communities” (HAALSI) research project.^25,26^ The Ukonga ward is a densely populated peri-urban area in the Ilala District of the Dar es Salaam Region. It is one of two wards covered by the Dar Es Salaam Health and Demographic Surveillance System that is also known as the Dar es Salaam Urban Cohort Study (DUCS).^27^ The DUCS aims to collect information from all ward residents. Data have been updated up to two times a year since the baseline census in 2011/12. According to 2019/20 DUCS data, a share of 21.3% (14,452 of 67,832) women were 40+ years old in Ukonga.

### Study population

Based on DUCS demographic data, 2400 women aged 40 years or older were randomly selected for a cross-sectional study on chronic health conditions. A total number of 1540 women could be interviewed at their home. Reasons for non-participation included repeated unsuccessful attempts to reach women at home and insufficient time to participate in the study. Twelve women participating in the study were unable or not willing to respond to questions about their literacy, education, marital status, number of children, religion, country of origin, or work status. These were excluded from the analysis, resulting in a final sample size of 1528 women aged 40 years or older. Half (761 of 1528) of the women in the study sample were randomly selected and invited to participate in point-of-care blood glucose and hemoglobin testing. Point-of-care test results could be obtained from 686 and 685 women, respectively. Reasons for non-participation in point-of-care testing included concerns about confidentiality, perceiving the request as too invasive, and religious reasons.

### Data collection

Field workers conducted computer-assisted personal interviews with the study participants. The interview included adapted versions of pre-existing screening instruments for angina pectoris (Rose Angina Questionnaire), depression (CES-D-10 scale), alcohol dependence (CAGE questionnaire), and cognitive impairment (US HRS cognitive test battery). Height, weight, and blood pressure were measured. Point-of-care blood testing used the CareSens Blood Glucose Monitoring System and the HemoCue Hemoglobin 201+ Analyzer.

### Outcomes

The main study outcomes were patterns of comorbidity and the extent of multimorbidity. Following others, we defined comorbidity as the presence of a chronic condition given the presence of an index condition, multimorbidity as the presence of 2+ chronic conditions, and discordant multimorbidity as the presence of chronic conditions in 2+ health areas.^28^ Comorbidity patterns were assessed by studying how often women with an index condition were affected by other chronic conditions. The extent of multimorbidity was assessed by studying, first, the frequency of 2+ chronic conditions, second, the frequency of chronic conditions in 2+ health areas, and third, the number of chronic conditions present.

### Chronic conditions

Conditions were chosen to mirror recent and projected leading causes of death and causes of disease burden among older women globally and in the WHO AFRO region, respectively.^29, 30^ Fifteen chronic conditions in three health areas were assessed:

• 10 physical health conditions: anemia, chronic cough, diabetes, high cholesterol, hypertension, ischemic heart disease, kidney disease, obesity, stroke, and underweight.
• 3 mental health conditions: signs of depression, cognitive problems, and alcohol problems.
• 2 infectious diseases: HIV infection and chronic tuberculosis (TB) infection.

Anemia, diabetes, hypertension, and over-/underweight were defined based on thresholds for hemoglobin, blood glucose, blood pressure, and the body mass index, respectively. The hemoglobin threshold was adjusted for smoking and African origin.^31^ Ischemic heart disease included a previous diagnosis of heart disease or heart failure or reporting symptoms of angina pectoris in the survey. Angina pectoris symptoms were assessed using a modified Rose Angina Questionnaire.^32,33^ Signs of depression were assessed with the 10-item Centre for Epidemiological Studies Depression (CES-D-10) scale and assumed for a CES-D-10 score of 10 or higher.^34^ A CAGE questionnaire score of 2 or higher was interpreted as signs of alcohol problems. Signs of cognitive problems were assessed based on recall tests (adapted from US Health and Retirement Study^35,36^) and self-rated memory. The presence of other chronic conditions was based on self-reporting either a prior diagnosis of the chronic condition or ever being treated for the chronic condition. Supplementary Table S1 provides detailed information about how chronic conditions were assessed.

### Data analysis

To analyse comorbidity patterns, we estimated the conditional prevalence of a chronic condition given the presence of another chronic condition as an index condition for each of the chronic conditions assessed. Findings were illustrated in a heatmap. Combinations of chronic conditions that were not observed in the study sample were reported with a zero prevalence. The prevalence of multimorbidity was estimated as the share of women affected by two or more of the assessed chronic conditions. The prevalence of discordant multimorbidity was estimated as the share of women with chronic conditions in two or more of the assessed health areas. Prevalences were reported with logit-transformed confidence intervals. The number of chronic conditions was determined by counting how many chronic conditions women had. Multivariable logit and linear regression models were used to assess the relationship of the multimorbidity prevalences and the number of chronic conditions present with sociodemographic characteristics, ever having smoked, and childhood health (measured on a 5-point Likert scale from very bad [−2] to very good [+2]). Estimations used 100 multiple imputations by chained equations for missing data. All analyses were conducted in Stata SE 15.1.

### Ethical considerations

Ethical approval was received from the Institutional Review Boards of Muhimbili University of Health and Allied Sciences, Tanzania, (2015-04-22/AEC/Vol.IX/82) and Harvard T.H. Chan School of Public Health, USA (14-4282). Participants gave written informed consent to participate in the study before interview and, where applicable, again for blood collection and testing.

## Results

### Sample characteristics

About half (52.9%) of the women in the study were aged 40–49 years, 26.7% were aged 50–59 years, and 20.4% were aged 60 years or older. Regarding their health history, most women (≥96.2%) never smoked and three of four regarded their childhood health as good or very good. Somewhat more women were Muslims (54.4%) than Christians (45.6%) and almost all (99.0%) were born in Tanzania. Most women were married or cohabitant (63.0%) and had children (97.7%). Four of five (80.1%) women reported to be able to read, write, or both. Slightly more than four of five (82.1%) women had not completed any formal education, that is, attended no or less than 7 years of school. Asked about what best describes the current work status, 64.1% of the women responded with homemaker and 21.0% with working part time or full time. A share of 14.9% of women were in retirement, on sick leave, or had a disability and classified as not working. One in two women (51.9%) reported not having had food in the house at least once during the past year, and 14.1% experienced this situation more than 10 times during the past year. Women who were randomly selected for point-of-care tests and those who were not appeared similar in these characteristics (Table 1).

**Table 1.** Sociodemographic characteristics of women in study sample.

	Total n (%)	Selected for point-of-care blood tests	
		No n (%)	Yes n (%)
	N = 1528	N = 767	N = 761
Age (40–44 years)	469 (30.7%)	231 (30.1%)	238 (31.3%)
45–49 years	339 (22.2%)	170 (22.2%)	169 (22.2%)
50–54 years	237 (15.5%)	121 (15.8%)	116 (15.2%)
55–59 years	171 (11.2%)	83 (10.8%)	88 (11.6%)
60–64 years	131 (8.6%)	63 (8.2%)	68 (8.9%)
65–69 years	80 (5.2%)	38 (5.0%)	42 (5.5%)
70+ years	101 (6.6%)	61 (8.0%)	40 (5.3%)
Ever smoked (no)	1,470 (96.2%)	737 (96.1%)	733 (96.3%)
Yes	50 (3.3%)	25 (3.3%)	25 (3.3%)
Missing	8 (0.5%)	5 (0.7%)	3 (0.4%)
Childhood health (very bad)	1 (0.1%)	1 (0.1%)	0 (0.0%)
Bad	24 (1.6%)	13 (1.7%)	11 (1.4%)
Moderate	313 (20.5%)	156 (20.3%)	157 (20.6%)
Good	863 (56.5%)	422 (55.0%)	441 (58.0%)
Very good	305 (20.0%)	163 (21.3%)	142 (18.7%)
Missing	22 (1.4%)	12 (1.6%)	10 (1.3%)
Religion (Islam)	831 (54.4%)	418 (54.5%)	413 (54.3%)
Christianity	697 (45.6%)	349 (45.5%)	348 (45.7%)
Country of origin (Tanzania)	1,513 (99.0%)	759 (99.0%)	754 (99.1%)
Other	15 (1.0%)	8 (1.0%)	7 (0.9%)
Marital status (married or cohabitant)	962 (63.0%)	475 (61.9%)	487 (64.0%)
Widowed	347 (22.7%)	184 (24.0%)	163 (21.4%)
Separated/Deserted	92 (6.0%)	47 (6.1%)	45 (5.9%)
Divorced	69 (4.5%)	32 (4.2%)	37 (4.9%)
Never married	58 (3.8%)	29 (3.8%)	29 (3.8%)
Children (none)	50 (3.3%)	25 (3.3%)	25 (3.3%)
1–2	372 (24.3%)	179 (23.3%)	193 (25.4%)
3–4	639 (41.8%)	315 (41.1%)	324 (42.6%)
5+	467 (30.6%)	248 (32.3%)	219 (28.8%)
Literacy (cannot read nor write)	305 (20.0%)	165 (21.5%)	140 (18.4%)
Can read and/or write	1,223 (80.0%)	602 (78.5%)	621 (81.6%)
Formal education completed (none)	1,255 (82.1%)	634 (82.7%)	621 (81.6%)
Primary (7–10 years)	56 (3.7%)	24 (3.1%)	32 (4.2%)
Secondary or more (10+ years)	217 (14.2%)	109 (14.2%)	108 (14.2%)
Work status (homemaker)	980 (64.1%)	493 (64.3%)	487 (64.0%)
Working	321 (21.0%)	151 (19.7%)	170 (22.3%)
Not working	227 (14.9%)	123 (16.0%)	104 (13.7%)
No food in house in past year (never)	735 (48.1%)	366 (47.7%)	369 (48.5%)
Rarely (once or twice)	447 (29.3%)	220 (28.7%)	227 (29.8%)
Sometimes (3–10 times)	131 (8.6%)	66 (8.6%)	65 (8.5%)
Often (more than 10 times)	215 (14.1%)	115 (15.0%)	100 (13.1%)

No significant differences between groups; Pearson's chi-squared tests, all *P* > 0.12.

### Prevalence of chronic conditions

Nearly all women had at least one chronic condition (93.7, 95% CI 92.3 to 95.2). The five most prevalent chronic conditions included four physical health conditions and one mental health condition: hypertension (estimated prevalence of 49.8%, 95% CI 47.2 to 52.3), obesity (39.9%, 95% CI 37.3 to 42.4), anemia (36.9%, 95% CI 33.3 to 40.5), signs of depression (32.5%, 95% CI 30.2 to 34.9), and diabetes (30.9%, 95% CI 27.6–34.2). The other assessed chronic conditions were found in less than 15% of the surveyed women and were in descending order: ischemic heart disease, signs of cognitive problems, HIV, high cholesterol, signs of alcohol problems, tuberculosis, stroke, underweight, chronic cough, and kidney impairment (Table 2 and Supplementary Figure S1).

**Table 2.** Prevalence of chronic conditions and multimorbidity among middle-aged and older women in peri-urban Tanzania.

	Observed prevalence % (n / N)	Estimated prevalence % (95% CI)
	N ≤ 1528	N = 1528
Any chronic condition	93.1 (630/677)	93.7 (92.3 to 95.2)
Any physical health conditions	89.4 (610/682)	89.9 (88 to 91.8)
Hypertension	49.7 (739/1,488)	49.8 (47.2 to 52.3)
Obesity	42.7 (652/1,528)	39.9 (37.3 to 42.4)
Anemia	36.4 (249/685)	36.9 (33.3 to 40.5)
Diabetes	30.6 (210/686)	30.9 (27.6 to 34.2)
Ischemic heart disease	14.4 (220/1,524)	14.4 (12.7 to 16.2)
High cholesterol	5.7 (86/1,520)	5.7 (4.5 to 6.8)
Stroke	5.0 (76/1,524)	5.0 (3.9 to 6.1)
Underweight	3.5 (54/1,528)	4.0 (3.0 to 5.0)
Chronic cough	3.6 (55/1,528)	3.6 (2.7 to 4.6)
Kindey impairment	3.2 (49/1,519)	3.2 (2.3 to 4.1)
Any mental health conditions	39.4 (595/1,509)	39.7 (37.2 to 42.1)
Signs of depression	32.4 (491/1,515)	32.5 (30.2 to 34.9)
Signs of cognitive problems	6.8 (104/1,521)	6.9 (5.6 to 8.2)
Signs of alcohol problems	5.3 (80/1,520)	5.3 (4.1 to 6.4)
Any infectious disease	10.7 (162/1,517)	10.7 (9.2 to 12.3)
HIV	6.6 (100/1,519)	6.6 (5.4 to 7.9)
Tuberculosis	5.2 (79/1,521)	5.2 (4.1 to 6.3)
Number of chronic conditions (none)	6.9 (47/677)	6.3 (4.8 to 7.7)
1	19.8 (134/677)	20 (17.6 to 22.3)
2+	73.3 (496/677)	73.8 (71.2 to 76.3)
Number of physical health conditions (none)	10.6 (72/682)	10.1 (8.2 to 12.0)
1	28.6 (195/682)	28.8 (26.1 to 31.5)
2+	60.9 (415/682)	61.1 (58.2 to 64.0)
Number of mental health conditions (none)	60.6 (914/1,509)	60.3 (57.9 to 62.8)
1	34.9 (526/1,509)	34.8 (32.4 to 37.2)
2+	4.6 (69/1,509)	4.9 (3.8 to 6.0)
Number of infectious diseases (none)	89.3 (1,355/1,517)	89.3 (87.7 to 90.8)
1	9.6 (145/1,517)	9.6 (8.1 to 11.1)
2	1.1 (17/1,517)	1.1 (0.6 to 1.6)
Number of impaired health areas (none)	6.9 (47/677)	6.3 (4.8 to 7.7)
1	51.7 (350/677)	52.1 (49.4 to 54.8)
2	36.9 (250/677)	36.8 (34.3 to 39.3)
3	4.4 (30/677)	4.9 (3.8 to 6.0)

N = 1528 after multiple imputation of missing data.

### Patterns of comorbidity

**Figure 1.**
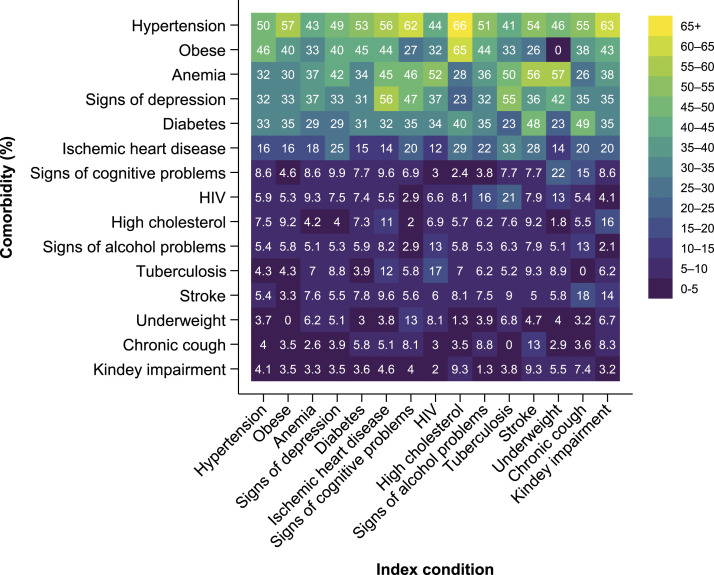
Comorbidity patterns among middle-aged and elderly women in peri-urban Tanzania. N = 1528 after multiple imputation of missing data. Off-diagonal values represent the conditional prevalence of a comorbidity given the presence of the index condition. Top-left to bottom-right diagonal values represent the unconditional prevalence of the chronic condition. Comorbidity patterns by age-group are provided in Supplementary Figure S1. Data with 95% confidence intervals are provided in Supplementary Table S2.

Among women aged 40 years or older, hypertension was the most prevalent comorbidity to several index conditions, with prevalence rates between 41.1% (95% CI 30.0 to 52.1) as a comorbidity to TB and 66.3% (95% CI 56.3 to 76.3) as a comorbidity to high cholesterol. Other comorbidities that were commonly present in more than 30% of the women who had one of the assessed chronic conditions as an index condition were obesity, anemia, signs of depression, and diabetes (Figure 1, upper 5 rows). Among the comorbidities that we observed less often overall, signs of cognitive problems were a comorbidity for 22.4% (95% CI 11.3 to 33.6) of women with underweight and 15.4% (95% CI 5.7 to 25.1) of women with chronic, non-TB-related cough. Signs of alcohol problems, in turn, appeared in 12.6% (95% CI 6.0 to 19.2) of women with HIV, in 12.6% (95% CI 3.9 to 21.4) of women with chronic, non-TB-related cough, and in 8.2% (95% CI 4.5 to 11.8) of women with ischemic heart disease. TB was most commonly a comorbidity among women with HIV (16.8%, 95% CI 9.5 to 24.1), followed by women with ischemic heart disease (11.9%, 95% CI 7.6 to 16.2) (Figure 1, lower 9 rows). Comparing comorbidity patterns across age groups, hypertension, obesity, anemia, and signs of depression were commonly present in more than 30% of 40–49 year-old women with an index condition. The prevalence of several of these common comorbidities increased among women aged 50 years or older. In addition, diabetes was commonly present in more than 30% of the women aged 50 years or older. Ischemic heart disease was commonly present in more than 20% of the women aged 50 years or older as well as in 40–49 year-old women with a medical history of high cholesterol (34%), TB (27%), or stroke (25%). In the oldest age group of women aged 60 years or older, signs of cognitive problems increased substantially as a comorbidity as well as a morbidity on their own (Supplementary Figure S1).

### Prevalence of multimorbidity

Three of four women aged 40 years or older were multimorbid (73.8%, 95% CI 71.2 to 76.3). Multiple physical chronic conditions affected 61.1% (95% CI 58.2 to 64.0) of women. About one of 20 women (4.9%, 95% CI 3.8 to 6.0) suffered from two or more mental health conditions and 1.1% (95% CI 0.6 to 1.6) were affected by TB and HIV. Discordant multimorbidity was present in 41.7% (95% CI 39.1 to 44.2) of the women in the study (Table 2).

**Figure 2.**
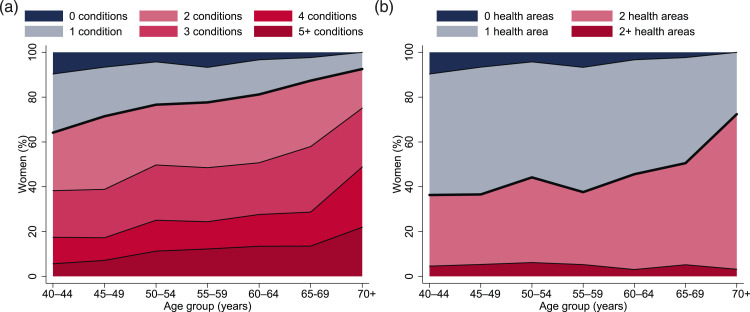
Extent of multimorbidity among middle-aged and elderly women in peri-urban Tanzania. (a) Number of chronic conditions. (b) Number of affected health areas. N = 1528 after multiple imputation of missing data. Red areas indicate (a) multimorbidity, that is, being affected by 2+ chronic conditions, and (b) discordant multimorbidity, that is, chronic conditions in 2+ health areas. Data is provided in Supplementary Tables S3(a)–(b).

Multimorbidity affected 64.1% (95% CI 59.3 to 69.0) of the women aged 40–44 years and 92.6% (95% CI 86.7 to 98.4) of the women aged 70 years or older. More than half of the women aged 50 years or older (54.1%, 95% CI 49.8 to 58.3) were affected by 3+ chronic conditions. Among the women aged 70 years or older, about three in four (75.1%, 95% CI 64.7 to 85.5) were affected by 3+ chronic conditions and one in five (21.9%, 95% CI 12.3 to 31.5) was affected by 5+ chronic conditions (Figure 2(a)). The prevalence of discordant multimorbidity was 36.3% (95% CI 31.8 to 40.8) among women aged 40–44 years and 72.4% (95% CI 63.3 to 81.5) among women aged 70 years or older (Figure 2(b)).

### Relationship of multimorbidity with age and other factors

In multivariable regressions, the prevalence of multimorbidity and the number of chronic conditions present were associated with age. The prevalence of discordant multimorbidity only increased for the oldest women in comparison to women aged 40–44 years. Adjusting for other sociodemographic characteristics, ever having smoked, and childhood health, women aged 50 years or older were more likely to be affected by multimorbidity than women aged 40–44 years. The oldest women in the study sample were 4.1 (95% CI 1.5 to 10.9) times more likely to be affected by multimorbidity and 2.3 (95% CI 1.3 to 4.3) times more likely to be affected by discordant multimorbidity than women aged 40–44 years. On average, women aged 70 years or older had 0.7 (95% CI 0.3 to 1.2) more chronic conditions than women aged 40–44 years.

Other factors in the multivariable regressions that were associated with a higher prevalence of multimorbidity and a higher number of chronic conditions present include worse childhood health, being widowed, not working, and higher food insecurity in the household. Better self-reported childhood health significantly lowered the odds of being affected by multimorbidity, the number of affected health areas, and the number of present chronic conditions. Being widowed, not working, and higher food insecurity in the household, in turn, were associated with more frequent and more extensive multimorbidity (Table 3).

**Table 3.** Relationship of multimorbidity and number of chronic conditions present with age and other factors among middle-aged and elderly women in peri-urban Tanzania

Covariate (reference group)	Affected by multimorbidity OR (95% CI)	Affected by discordant multimorbidity OR (95% CI)	Number of chronic conditions Coef. (95% CI)
Age (40–44 years)	1	1	0
45–49 years	1.4 (1.0 to 2.0)	1.0 (0.7 to 1.3)	0.1 (−0.1 to 0.3)
50–54 years	1.8 (1.2 to 2.8)^**^	1.4 (1.0 to 2.0)	0.4 (0.1 to 0.6)^**^
55–59 years	2.0 (1.2 to 3.2)^**^	1.0 (0.7 to 1.5)	0.4 (0.1 to 0.7)^**^
60–64 years	2.1 (1.2 to 3.8)^*^	1.1 (0.7 to 1.8)	0.4 (0.1 to 0.7)^*^
65–69 years	3.0 (1.2 to 7.5)^*^	1.2 (0.7 to 2.0)	0.4 (−0.1 to 0.8)
70+ years	4.1 (1.5 to 10.9)^**^	2.3 (1.3 to 4.3)^**^	0.7 (0.3 to 1.2)^***^
Ever smoked (no)	1	1	0
Yes	1.6 (0.5 to 5.0)	2.0 (1.0 to 4.2)	0.4 (0 to 0.9)
Childhood health (very bad–very good)	0.7 (0.6 to 0.9)^***^	0.5 (0.5 to 0.6)^***^	−0.3 (−0.4 to −0.1)^***^
Religion (Islam)	1	1	0
Christianity	0.9 (0.7 to 1.2)	1.1 (0.8 to 1.4)	0.03 (−0.2 to 0.1)
Country of origin (Tanzania)	1	1	0
Other	0.8 (0.2 to 3.1)	1.1 (0.3 to 3.8)	0.03 (−0.8 to 0.8)
Marital status (married or cohabitant)	1	1	0
Widowed	1.6 (1.0 to 2.6)^*^	2.0 (1.5 to 2.8)^***^	0.4 (0.2 to 0.6)***
Separated/Deserted	1.0 (0.6 to 1.9)	1.1 (0.7 to 1.8)	0.1 (−0.3 to 0.4)
Divorced	1.2 (0.6 to 2.5)	1.3 (0.7 to 2.2)	0.2 (−0.1 to 0.6)
Never married	2.0 (0.9 to 4.7)	1.7 (0.9 to 3.4)	0.2 (−0.3 to 0.6)
Children (none)	1	1	0
1–2	1.1 (0.5 to 2.8)	1 (0.5 to 2.0)	0.3 (−0.2 to 0.8)
3–4	1.0 (0.4 to 2.4)	0.9 (0.5 to 1.8)	0.2 (−0.3 to 0.6)
5+	0.8 (0.3 to 2.1)	0.9 (0.4 to 1.7)	0.03 (−0.4 to 0.5)
Literacy (cannot read nor write)	1	1	0
Can read and/or write	1.0 (0.6 to 1.5)	1.0 (0.7 to 1.4)	−0.1 (−0.3 to 0.2)
Formal education completed (none)	1	1	0
Primary (7–10 years)	1.3 (0.6 to 2.8)	1.6 (0.8 to 2.9)	0.5 (0.1 to 1.0)^**^
Secondary or more (10+ years)	0.9 (0.6 to 1.3)	1.0 (0.7 to 1.4)	−0.1 (−0.3 to 0.2)
Work status (homemaker)	1	1	0
Working	1.3 (1.0 to 1.9)	2.0 (1.5 to 2.7)^***^	0.1 (−0.1 to 0.3)
Not working	1.9 (1.1 to 3.1)^*^	2.3 (1.7 to 3.3)^***^	0.5 (0.2 to 0.7)^***^
No food in house in past year (never)	1	1	0
Rarely (once or twice)	1.2 (0.8 to 1.6)	1.2 (0.9 to 1.7)	0.1 (−0.1 to 0.3)
Sometimes (3–10 times)	2.1 (1.1 to 4.0)^*^	1.8 (1.1 to 2.7)^*^	0.4 (0.1 to 0.8)^**^
Often (more than 10 times)	1.8 (1.1 to 2.8)^*^	3.4 (2.3 to 4.9)^***^	0.4 (0.2 to 0.7)^***^
Household wealth (bottom 20%)	1	1	0
20%–40%	0.9 (0.5 to 1.3)	0.8 (0.5 to 1.1)	−0.01 (−0.3 to 0.2)
40%–60%	0.9 (0.5 to 1.4)	0.8 (0.5 to 1.2)	0.04 (−0.2 to 0.3)
60%–80%	1.0 (0.6 to 1.7)	0.9 (0.6 to 1.3)	0.1 (−0.1 to 0.4)
Top 20%	1.0 (0.6 to 1.7)	0.9 (0.6 to 1.3)	0.1 (−0.2 to 0.4)
Constant	2.1 (0.7 to 6.1)	0.6 (0.3 to 1.4)	2.0 (1.5 to 2.6)^***^

N = 1528 after multiple imputation of missing data. *P < 0.05, **P < 0.01, ***P < 0.001.

## Discussion

We studied comorbidity patterns and the extent of multimorbidity among women aged 40 years or older in the peri-urban Ukonga ward of Dar es Salaam, Tanzania. Nine of ten women in the study sample were affected by at least one chronic condition. Three of four women were affected by multimorbidity, and four of ten women were affected by discordant multimorbidity. Hypertension, obesity, anemia, signs of depression, diabetes, and ischemic heart disease were the most prevalent chronic conditions as well as comorbidities. In multivariable analyses, age, childhood health, being widowed, not working, and experiencing food insecurity were associated with the prevalence of multimorbidity, the prevalence of discordant multimorbidity, and the number of chronic conditions present.

A scoping review of multimorbidity in low- and middle-income countries reported a multimorbidity prevalence ranging from 3.2% to 67.8% among adults aged 18 or older, from 19.4% to 80% among adults aged 40 or older, and from 27.3% to 90.5% among adults aged 60 or older; women (25% to 52.2%) were more often affected by multimorbidity than men (13.4% to 38.6%).^37^ Prior studies in Sub-Saharan African countries found a multimorbidity prevalence of 28.7% among adults aged 40–60 years in urban Kenya,^38^ 48.3% among adults aged 60 or older in Ghana,^20^ 65% among adults aged 60 or older in urban Burkina Faso,^39^ and 69.4% among adults aged 40 or older in rural South Africa.^40^ A study of men and women aged 40 years or older in the Ukonga ward in Dar es Salaam, Tanzania, found a prevalence of multimorbidity and discordant multimorbidity of 25.3% and 2.5%, respectively.^16^ Differences in the age and gender compositions of the study populations, the study settings, as well as differing assessments of chronic conditions and definitions of multimorbidity, however, limit the comparability of findings across studies.

The prevalence of multimorbidity increased continuously with age among the women in our study sample. The prevalence of discordant multimorbidity increased for women aged 70 years or older. The number of chronic conditions present increased stepwise with age and plateaued for some time between 50 and 69 years of age. In contrast to our findings, studies of multimorbidity in middle- and high-income countries found that multimorbidity increased at a similar or a declining rate above the age of 70 years.^20,41^ In addition to age, we found worse childhood health, being widowed or not working, and lacking food in the household to be associated with multimorbidity. We found no significant association of the level of education or household wealth with multimorbidity. Previous studies of multimorbidity in low- and middle-income countries reported inconsistent findings about the association of wealth with multimorbidity.^37^ Similarly, lower education increased the odds for multimorbidity in some studies, whereas no significant association was found in other studies.^18,20^ We studied women in one peri-urban area of Dar Es Salaam. Therefore, wealth and education levels were rather similar among the women in the study sample. The negative associations of multimorbidity with food insecurity, being widowed, and not working, however, suggest that multimorbidity was affected by social determinants.

The five most prevalent chronic conditions and comorbidities among the women in our study were hypertension, obesity, anemia, signs of depression, and diabetes (>30% each). Hypertension was the most prevalent chronic condition (49.8%) and the most common comorbidity (41.1% to 66.3%). A multi-country study that included data from adults aged 50 years or older from Ghana and South Africa found hypertension to be the most common comorbidity, especially among people affected by obesity, stroke, diabetes, and angina.^20^ Similar results were described by another study from South Africa^40^ and a scoping review of studies in low- and middle-income countries.^37^ Obesity was the second most prevalent chronic condition (39.9%), but the used global BMI cut-off for obesity might overestimate body fat and health risks in black populations.^42,43^ Anemia was the third most prevalent chronic condition (36.9%). A previous study of anemia in 27 nationally representative samples of women aged 15 to 49 years in Sub-Saharan Africa reported that 44.1% (18,438 of 41,809) of women aged 35–44 years and 43.1% (6,267 of 14,542) of women aged 45–49 years were anemic.^44^ Signs of depression (32.5%) were the fourth most common chronic condition. A meta-analysis of 23 depression studies conducted in African countries estimated a pooled prevalence of depression of 43.1% among elderly women and of 30.9% among elderly men.^45^ Studies that used a screening tool, like the CES-D, to measure depression estimated a higher prevalence (43.1%) than studies that used a diagnostic tool (24.2%).^45^ Diabetes was the fifth most common chronic condition among elderly women in the Ukonga ward. A study using random point-of-care measures of glycosylated hemoglobin in rural Tanzania reported a diabetes prevalence (HbA1c ≥ 6.5%) of 14.8% (95% CI 10.6 to 20.2) in adults aged 40–49 years and 26.0% (95% CI 19.5 to 33.8) in adults aged 50 years or older.^46^ This prevalence of diabetes was considered higher than in several other studies in the region.^46^ Our estimate suggests that the prevalence of diabetes in urban women aged 40 years or older could be as high as 30.9%.

Overall, chronic non-communicable diseases and multimorbidity involving non-communicable diseases were common among the women participating in our study. At the same time, various studies conducted over the past decade indicate that Tanzanian health facilities have gaps in providing ongoing or acute care for non-communicable diseases.^47–53^ Identified gaps include a lack of guidelines for the management of non-communicable diseases and lower experience of staff in managing non-communicable diseases as compared to HIV.^47^ For instance, a study of hypertension diagnosis and treatment in Dar Es Salaam reported that only 58% of women aged 40 years or older were aware of their diagnosis, 28% were treated, and 14% had controlled blood pressure.^54^ Our findings on the comorbidity patterns and extent of multimorbidity among women in peri-urban Dar Es Salaam provide information that can help assess health care and prevention needs. In addition, the presented comorbidity patterns can be used to purposively screen women who visit a health facility with an index condition for common and potentially underdiagnosed comorbidities.

This study has several strengths and limitations. Strengths of this study include its random sampling of women aged 40 years or older from the Ukonga ward population. Our study exceeded a suggested minimum number of twelve chronic conditions^41^ and included three health areas (physical non-communicable diseases of long duration, mental health conditions of long duration, and infectious conditions of long duration) as has been suggested for multimorbidity assessment.^28^ Further, nine chronic conditions were assessed based on body measurements, weight, blood testing, screening instruments, and only six relied on self-report. The study’s limitations include a non-response rate of 35.8% (860 of 2400). Reasons for non-participation besides unsuccessful attempts of home visits and insufficient time for participation remain unclear. Some screening instruments were adapted and/or lacked validation in the used language and population. Further, we included risk factors into the assessed chronic conditions (e.g., hypertension and high cholesterol). Although risk factors have a smaller impact on health than diseases,^55^ we decided to include them in our multimorbidity assessment as knowledge of having a risk factor or being on treatment for one can add to the disease burden. Finally, common chronic non-communicable conditions such as musculoskeletal conditions, diseases of the nervous system and neoplasms, have not been assessed.

## Conclusion

Middle-aged and elderly women in the peri-urban Ukonga ward of Dar es Salaam, Tanzania, were extensively affected by multimorbidity, including the joint occurrence of chronic physical conditions, mental health conditions, and/or infectious diseases. The high prevalence of multimorbidity in the assessed, general population of middle-aged and elderly women suggests a substantial need for multimorbidity care in Tanzania. The presented comorbidity patterns can guide multimorbidity screening and help identify health care and prevention needs.

## Supplemental Material

sj-pdf-1-cob-10.1177_26335565221076254 – Supplemental Material for Patterns of comorbidity and multimorbidity among middle-aged and elderly women in peri-urban Tanzania Click here for additional data file. Supplemental Material, sj-pdf-1-cob-10.1177_26335565221076254 for Patterns of comorbidity and multimorbidity among middle-aged and elderly women in peri-urban Tanzania by Laura-Marie Stieglitz, Till Bärnighausen, Germana H. Leyna, Patrick Kazonda, Japhet Killewo, Julia K. Rohr and Stefan Kohler in Journal of Comorbidity
