# Supplementary material for: Patterns of comorbidity and multimorbidity among middle-aged and elderly women in peri-urban Tanzania
Source: J Multimorb Comorb. 2022 Feb 22;12:26335565221076254. doi: 10.1177/26335565221076254 (PMC9106316; doi:10.1177/26335565221076254)
Supplement: sj-pdf-1-cob-10.1177_26335565221076254 – Supplemental Material for Patterns of comorbidity and multimorbidity among middle-aged and elderly women in peri-urban Tanzania [file sj-pdf-1-cob-10.1177_26335565221076254.pdf]

**Supplementary material**

**Table S1:** Assessment and definition of chronic conditions

| Chronic condition | Assessment | Definition |
|---|---|---|
| Anemia | 1. Hemoglobin point-of-care test<br>2. Self-report of smoking | Hb < 12 mg/dl; threshold adjustments for smoking (+0.3 mg/dl) and African origin (−1 mg/dl) |
| Signs of alcohol problems | CAGE questionnaire for problem drinking and potential alcohol problems | CAGE score ≥ 2 |
| Signs of cognitive problems | 1. Self-rated present memory<br>2. Immediate and delayed word recall of 10 nouns, date, and president naming; scoring of correct answers; adapted from US Health and Retirement Study | Present memory rated as fair or poor, score ≤ 1.5 standard deviation of population mean |
| Chronic cough, no TB | Self-reported coughing | Self-report of usually coughing and not having TB as a chronic condition |
| Signs of depression | 10-item Centre for Epidemiological Studies Depression Scale (CES-D-10) | CES-D-10 score ≥ 10 |
| Diabetes | 1. Blood glucose point-of-care test<br>2. Self-report of current treatment | Blood glucose ≥ 200 mg/dl, fasting blood glucose ≥ 126 mg/dl, or currently on treatment for diabetes with diet, weight loss, pills, or insulin injection |
| Kidney disease | Self-reported diagnosis | Ever diagnosed with kidney disease or low kidney function |
| HIV | Self-reported diagnosis | Ever tested HIV positive |
| High cholesterol | Self-reported diagnosis | Ever diagnosed with high cholesterol |
| Hypertension | 1. Blood pressure was measured up to three times, between the second and the third time was a delay of two minutes; the mean of all measurements was calculated<br>2. Self-report of current treatment | Systolic pressure ≥ 140 mmHg, diastolic pressure ≥ 90 mmHg or currently on treatment for hypertension |
| Ischemic heart disease | 1. Self-reported diagnosis of angina, heart failure, or heart attack<br>2. Modified Rose Angina Questionnaire | Rose Angina Questionnaire criteria, ever received diagnosis of angina, or ever received diagnosis of heart attack |
| Obesity | Measured weight and height; BMI was calculated | BMI > 30 $kg/m^2$ |
| Stroke | 1. Self-reported diagnosis<br>2. Self-reported treatment | Ever diagnosed with stroke, mini-stroke, transient ischemic attack; suddenly lost half vision, or ever received treatment for stroke |
| Tuberculosis (TB) | 1. Self-reported diagnosis<br>2. Self-reported treatment | Ever diagnosed with TB, but never received treatment, or currently on TB treatment |
| Underweight | Measured weight and height; BMI was calculated | BMI < 18.5 $kg/m^2$ |

Self-reported diagnoses were obtained by asking: Have you ever been told by a doctor, nurse, or other healthcare worker that you have had … ? Rose Angina Questionnaire criteria were: Experienced any pain or discomfort in the chest or pain going to the left arm or neck when walking uphill or hurrying during the last 12 months; stopping, slowing down, or carrying on after taking a pain-relieving medicine that dissolves in the mouth (e.g., a nitro spray or tablet) when getting pain or discomfort while walking; and pain or discomfort is relieved when standing still.

**Table S2:** Prevalence of comorbidities among middle-aged and elderly women in peri-urban Tanzania (women aged 40+ years)

| Prevalence % [95% CI] | Index condition | | | | | | | | | | | | | | |
|---|---|---|---|---|---|---|---|---|---|---|---|---|---|---|---|
| Comorbidity | Hyper-tension | Obese | Anemia | CES-D-10 score ≥10 | Diabetes | Ischemic heart disease | Cognitive problems | HIV | High chol-esterol | CAGE Score 2+ | Tuber-culosis | Stroke | Under-weight | Chronic cough (no TB) | Kidney disease |
| **Hypertension** | 49.8 (47.2 to 52.3) | 57.0 (53.0 to 61.0) | 43.3 (38.1 to 48.6) | 48.8 (44.3 to 53.2) | 52.9 (46.9 to 58.8) | 55.6 (49.0 to 62.2) | 62.1 (52.4 to 71.8) | 44.4 (34.6 to 54.1) | 66.3 (56.3 to 76.3) | 51.3 (40.3 to 62.2) | 41.1 (30.0 to 52.1) | 53.8 (42.6 to 65.1) | 45.9 (32.7 to 59.1) | 54.7 (41.5 to 67.9) | 63.3 (49.8 to 76.8) |
| **Anemia** | 45.6 (42.0 to 49.3) | 39.9 (37.3 to 42.4) | 32.7 (27.6 to 37.8) | 40.0 (35.6 to 44.5) | 44.6 (38.7 to 50.5) | 44.4 (37.8 to 51.1) | 26.6 (17.5 to 35.8) | 32.1 (22.9 to 41.3) | 64.6 (54.5 to 74.8) | 44.2 (33.2 to 55.1) | 32.9 (22.4 to 43.5) | 26.3 (16.2 to 36.4) | 0 | 38.4 (25.3 to 51.4) | 43.4 (29.4 to 57.3) |
| **Obese** | 32.1 (27.3 to 37.0) | 30.3 (25.1 to 35.5) | 36.9 (33.3 to 40.5) | 41.6 (35.2 to 48) | 34.4 (28.0 to 40.9) | 45.4 (35.9 to 54.8) | 46.0 (33.1 to 58.8) | 51.5 (38.5 to 64.6) | 27.5 (14.5 to 40.6) | 36.1 (22.6 to 49.6) | 49.9 (33.2 to 66.6) | 56.3 (40.5 to 72) | 56.9 (39.5 to 74.4) | 26.0 (7.7 to 44.3) | 37.7 (18.1 to 57.4) |
| **Signs of depression** | 31.9 (28.5 to 35.2) | 32.7 (28.9 to 36.5) | 36.6 (31.5 to 41.8) | 32.5 (30.2 to 34.9) | 30.5 (25.0 to 36.1) | 56.4 (49.8 to 62.9) | 46.7 (37 to 56.5) | 37 (27.5 to 46.5) | 23.3 (14.3 to 32.2) | 32.5 (22.2 to 42.8) | 55.0 (43.9 to 66) | 35.7 (24.9 to 46.5) | 41.8 (28.9 to 54.8) | 35.0 (22.3 to 47.6) | 35 (21.6 to 48.3) |
| **Diabetes** | 32.8 (28.2 to 37.4) | 34.6 (29.3 to 39.8) | 28.8 (23.5 to 34.2) | 29.0 (23.2 to 34.8) | 30.9 (27.6 to 34.2) | 32.1 (23.4 to 40.8) | 34.6 (22.5 to 46.7) | 34.3 (21.4 to 47.2) | 39.7 (26.5 to 52.9) | 34.8 (21.7 to 48) | 23.5 (7.4 to 39.5) | 48.3 (32.7 to 64) | 23.0 (8.5 to 37.5) | 49.1 (28.6 to 69.6) | 34.6 (15.3 to 53.8) |
| **Ischemic heart disease** | 16.1 (13.5 to 18.7) | 16.1 (13.1 to 19.0) | 17.8 (13.7 to 21.8) | 25.0 (21.2 to 28.8) | 15 (10.8 to 19.2) | 14.4 (12.7 to 16.2) | 20.1 (12.4 to 27.8) | 11.9 (5.6 to 18.2) | 29 (19.4 to 38.6) | 22.4 (13.3 to 31.6) | 33.0 (22.6 to 43.3) | 27.7 (17.6 to 37.7) | 13.6 (4.6 to 22.5) | 20.0 (9.4 to 30.6) | 20.3 (9.1 to 31.6) |
| **Cognitive impair-ment symptoms** | 8.6 (6.6 to 10.6) | 4.6 (2.8 to 6.4) | 8.6 (5.8 to 11.4) | 9.9 (7.2 to 12.6) | 7.7 (4.8 to 10.7) | 9.6 (5.7 to 13.5) | 6.9 (5.6 to 8.2) | 3 (−0.3 to 6.4) | 2.4 (−0.9 to 5.8) | 3.8 (−0.4 to 8.1) | 7.7 (1.8 to 13.6) | 7.7 (1.5 to 13.9) | 22.4 (11.3 to 33.6) | 15.4 (5.7 to 25.1) | 8.6 (0.6 to 16.5) |
| **HIV** | 5.9 (4.2 to 7.6) | 5.3 (3.5 to 7.1) | 9.3 (6.4 to 12.1) | 7.5 (5.2 to 9.9) | 7.4 (4.3 to 10.4) | 5.5 (2.5 to 8.5) | 2.9 (−0.3 to 6.1) | 6.6 (5.4 to 7.9) | 8.1 (2.3 to 13.9) | 15.9 (7.7 to 24.1) | 21.4 (12.4 to 30.4) | 7.9 (1.8 to 14.0) | 13.4 (4.7 to 22.1) | 5.4 (−0.5 to 11.4) | 4.1 (−1.5 to 9.8) |
| **High cholesterol** | 7.5 (5.6 to 9.4) | 9.2 (6.9 to 11.5) | 4.2 (2.1 to 6.4) | 4.0 (2.3 to 5.8) | 7.3 (4.5 to 10.1) | 11.4 (7.2 to 15.6) | 2 (−0.7 to 4.7) | 6.9 (2 to 11.9) | 5.7 (4.5 to 6.8) | 6.2 (0.9 to 11.5) | 7.6 (1.7 to 13.4) | 9.2 (2.7 to 15.7) | 1.8 (−1.7 to 5.2) | 5.5 (−0.5 to 11.5) | 16.2 (5.9 to 26.5) |
| **Signs of alcohol problems** | 5.4 (3.8 to 7.0) | 5.8 (4.0 to 7.7) | 5.1 (3.0 to 7.3) | 5.3 (3.3 to 7.2) | 5.9 (3.4 to 8.5) | 8.2 (4.5 to 11.8) | 2.9 (−0.3 to 6.2) | 12.6 (6.0 to 19.2) | 5.8 (0.9 to 10.7) | 5.3 (4.1 to 6.4) | 6.3 (1 to 11.6) | 7.9 (1.8 to 14.0) | 5.1 (−0.5 to 10.8) | 12.6 (3.9 to 21.4) | 2.1 (−2 to 6.2) |
| **Tuberculosis** | 4.3 (2.8 to 5.8) | 4.3 (2.7 to 5.9) | 7.0 (4.3 to 9.8) | 8.8 (6.3 to 11.3) | 3.9 (1.2 to 6.7) | 11.9 (7.6 to 16.2) | 5.8 (1.3 to 10.3) | 16.8 (9.5 to 24.1) | 7 (1.6 to 12.4) | 6.2 (0.9 to 11.5) | 5.2 (4.1 to 6.3) | 9.3 (2.7 to 15.9) | 8.9 (1.5 to 16.3) | 0 | 6.2 (−0.6 to 12.9) |
| **Stroke** | 5.4 (3.8 to 7.0) | 3.3 (1.9 to 4.7) | 7.6 (5.0 to 10.3) | 5.5 (3.5 to 7.5) | 7.8 (4.9 to 10.7) | 9.6 (5.7 to 13.5) | 5.6 (1 to 10.1) | 6.0 (1.3 to 10.6) | 8.1 (2.4 to 13.9) | 7.5 (1.7 to 13.3) | 9.0 (2.6 to 15.3) | 5 (3.9 to 6.1) | 5.8 (−0.5 to 12.2) | 18.2 (8 to 28.3) | 14.3 (4.5 to 24.1) |
| **Underweight** | 3.7 (2.3 to 5.1) | 0 | 6.2 (3.8 to 8.5) | 5.1 (3.1 to 7.2) | 3.0 (1.0 to 5.0) | 3.8 (1.1 to 6.4) | 13.0 (6.0 to 20.0) | 8.1 (2.7 to 13.4) | 1.3 (−1.2 to 3.7) | 3.9 (−0.4 to 8.2) | 6.8 (1.1 to 12.6) | 4.7 (−0.4 to 9.7) | 4.0 (3.0 to 5.0) | 3.2 (−2.3 to 8.7) | 6.7 (−0.5 to 14) |
| **Chronic cough, no TB** | 4.0 (2.6 to 5.4) | 3.5 (2.0 to 5.0) | 2.6 (0.7 to 4.5) | 3.9 (2.2 to 5.7) | 5.8 (3.0 to 8.5) | 5.1 (2.1 to 8) | 8.1 (2.8 to 13.5) | 3.0 (−0.3 to 6.3) | 3.5 (−0.4 to 7.4) | 8.8 (2.6 to 15) | 0 | 13.2 (5.6 to 20.9) | 2.9 (−2.1 to 8.0) | 3.6 (2.7 to 4.6) | 8.3 (0.5 to 16.1) |
| **Kidney disease** | 4.1 (2.7 to 5.5) | 3.5 (2.0 to 5.0) | 3.3 (1.4 to 5.2) | 3.5 (1.9 to 5.1) | 3.6 (1.4 to 5.8) | 4.6 (1.8 to 7.3) | 4 (0.2 to 7.9) | 2.0 (−0.8 to 4.8) | 9.3 (3.1 to 15.4) | 1.3 (−1.2 to 3.8) | 3.8 (−0.4 to 8.1) | 9.3 (2.7 to 15.8) | 5.5 (−0.5 to 11.4) | 7.4 (0.4 to 14.3) | 3.2 (2.3 to 4.1) |

N = 1528 after multiple imputation of missing data. Top-left to bottom-right diagonal values represent the unconditional prevalence of the chronic condition.

**Table S2:** Prevalence of comorbidities among middle-aged and elderly women in peri-urban Tanzania (continued for women aged 40–49 years)

| Prevalence % [95% CI] | Index condition | | | | | | | | | | | | | | |
|---|---|---|---|---|---|---|---|---|---|---|---|---|---|---|---|
| Comorbidity | Hyper-tension | Obese | Anemia | CES-D-10 score ≥10 | Diabetes | Ischemic heart disease | Cognitive problems | HIV | High chol-esterol | CAGE Score 2+ | Tuber-culosis | Stroke | Under-weight | Chronic cough (no TB) | Kidney disease |
| **Hypertension** | 38.0 (34.4 to 41.5) | 47.2 (41.4 to 52.9) | 32.4 (25.9 to 38.9) | 37.8 (31.2 to 44.3) | 40.3 (31.6 to 49.0) | 41.8 (30.7 to 52.8) | 52 (27 to 77) | 43.4 (30.6 to 56.1) | 50.1 (30.8 to 69.3) | 39.7 (24.1 to 55.3) | 41 (23.1 to 58.9) | 37.7 (20.9 to 54.5) | 31.4 (10.8 to 52) | 30.6 (11.7 to 49.4) | 50.2 (25.7 to 74.7) |
| **Anemia** | 49.9 (43.9 to 55.9) | 40.2 (36.6 to 43.8) | 33.1 (26.4 to 39.7) | 44 (37.3 to 50.7) | 43.5 (34.7 to 52.4) | 45.5 (34.3 to 56.6) | 40.3 (15.6 to 65) | 34.8 (22.5 to 47.2) | 57.7 (38.6 to 76.7) | 44.8 (29 to 60.7) | 35.1 (17.6 to 52.5) | 21.2 (6.5 to 35.9) | 0 | 52.8 (31.6 to 74) | 39.6 (14.9 to 64.3) |
| **Obese** | 34.2 (26.7 to 41.6) | 33.0 (25.7 to 40.3) | 40.1 (35.1 to 45.1) | 43.8 (34.7 to 52.8) | 39.6 (30.1 to 49.1) | 44.9 (31.2 to 58.6) | 37.8 (8.2 to 67.3) | 48.7 (33 to 64.3) | 31.4 (8.1 to 54.6) | 40.1 (20 to 60.2) | 54.3 (31.3 to 77.3) | 52.7 (31.3 to 74.1) | 60.7 (34.3 to 87.2) | 24.4 (1 to 47.9) | 49.5 (17.2 to 81.7) |
| **Signs of depression** | 29.1 (23.7 to 34.5) | 32.0 (26.6 to 37.4) | 31.9 (25.4 to 38.5) | 29.3 (26 to 32.6) | 26.9 (19.2 to 34.7) | 51.3 (40.2 to 62.4) | 42.1 (18.6 to 65.6) | 38.1 (25.6 to 50.6) | 26.8 (9.8 to 43.8) | 41.9 (26.2 to 57.7) | 47.9 (29.7 to 66.1) | 28.1 (12.5 to 43.7) | 30.5 (10.3 to 50.6) | 47.7 (27.3 to 68.2) | 43.7 (19.4 to 68) |
| **Diabetes** | 27.9 (20.9 to 34.8) | 28.4 (21.3 to 35.6) | 25.9 (19 to 32.8) | 24.2 (16.2 to 32.2) | 26.3 (21.6 to 30.9) | 24.6 (12.4 to 36.7) | 23.9 (−1.3 to 49.1) | 30.3 (14.7 to 46.0) | 22.7 (1.5 to 44.0) | 24.1 (6.7 to 41.4) | 21.3 (2.4 to 40.2) | 40.5 (19.6 to 61.4) | 19 (−1.2 to 39.2) | 39.3 (12.8 to 65.8) | 29.5 (0.2 to 58.8) |
| **Ischemic heart disease** | 11.7 (7.9 to 15.5) | 12 (8.3 to 15.8) | 11.9 (7.6 to 16.2) | 18.7 (13.4 to 23.9) | 10.0 (4.9 to 15) | 10.6 (8.4 to 12.9) | 0 | 10.2 (2.5 to 18.0) | 34.5 (16.2 to 52.7) | 15.7 (4.2 to 27.3) | 26.6 (10.8 to 42.5) | 24.9 (9.9 to 39.9) | 4.8 (−4.4 to 13.9) | 17.4 (1.9 to 32.9) | 18.7 (−0.4 to 37.8) |
| **Cognitive impair-ment symptoms** | 3.2 (1.1 to 5.4) | 2.4 (0.6 to 4.2) | 2.2 (0.2 to 4.2) | 3.4 (0.9 to 5.8) | 2.1 (−0.3 to 4.6) | 0 | 2.4 (1.2 to 3.5) | 1.7 (−1.6 to 5) | 0 | 0 | 3.3 (−3.1 to 9.7) | 0 | 5.3 (−4.7 to 15.2) | 0 | 0 |
| **HIV** | 9.1 (5.7 to 12.6) | 6.9 (4.0 to 9.9) | 9.7 (5.9 to 13.6) | 10.4 (6.3 to 14.6) | 9.3 (4.1 to 14.4) | 7.7 (1.8 to 13.6) | 5.8 (−5.3 to 16.8) | 8.0 (6.0 to 10.0) | 7.7 (−2.6 to 17.9) | 19.4 (6.6 to 32.2) | 30 (13.5 to 46.4) | 12.5 (1 to 23.9) | 33.5 (13.1 to 53.9) | 13 (−0.7 to 26.8) | 6.4 (−5.7 to 18.4) |
| **High cholesterol** | 4.7 (2.2 to 7.2) | 5.1 (2.6 to 7.6) | 2.8 (0.5 to 5.1) | 3.3 (0.9 to 5.6) | 3.1 (0 to 6.2) | 11.5 (4.4 to 18.6) | 0 | 3.4 (−1.2 to 8.1) | 3.6 (2.2 to 4.9) | 2.6 (−2.5 to 7.8) | 0 | 3.1 (−2.9 to 9.1) | 0 | 4.3 (−4 to 12.7) | 18.6 (−0.4 to 37.6) |
| **Signs of alcohol problems** | 5.4 (2.8 to 8.1) | 5.8 (3.1 to 8.5) | 5.2 (2.2 to 8.2) | 7.5 (3.9 to 11) | 4.8 (1.1 to 8.5) | 7.7 (1.8 to 13.6) | 0 | 12.6 (4 to 21.2) | 3.9 (−3.6 to 11.3) | 5.2 (3.6 to 6.8) | 6.7 (−2.3 to 15.6) | 12.6 (1 to 24.1) | 9.4 (−3.1 to 21.9) | 17.4 (1.9 to 33.0) | 6.3 (−5.7 to 18.3) |
| **Tuberculosis** | 4.4 (2.0 to 6.9) | 3.6 (1.4 to 5.7) | 5.6 (2.5 to 8.6) | 6.7 (3.3 to 10.1) | 3.3 (0.2 to 6.5) | 10.3 (3.5 to 17.0) | 5.8 (−5.3 to 16.8) | 15.3 (6.1 to 24.6) | 0 | 5.3 (−1.8 to 12.4) | 4.1 (2.7 to 5.5) | 3.1 (−2.9 to 9.2) | 9.6 (−3.1 to 22.3) | 0 | 12.6 (−3.7 to 28.9) |
| **Stroke** | 4.3 (1.9 to 6.8) | 2.3 (0.5 to 4.1) | 5.8 (2.7 to 8.8) | 4.2 (1.5 to 6.9) | 6.8 (2.6 to 10.9) | 10.2 (3.5 to 17.0) | 0 | 6.8 (0.4 to 13.3) | 3.8 (−3.5 to 11.2) | 10.6 (0.8 to 20.4) | 3.4 (−3.1 to 9.8) | 4.4 (2.9 to 5.9) | 5.4 (−4.8 to 15.6) | 8.7 (−2.8 to 20.2) | 12.5 (−3.7 to 28.6) |
| **Underweight** | 2.4 (0.5 to 4.3) | 0 | 4.4 (1.8 to 7) | 3.0 (0.7 to 5.4) | 2.1 (−0.3 to 4.5) | 1.3 (−1.2 to 3.8) | 6.5 (−5.8 to 18.8) | 12.1 (3.7 to 20.6) | 0 | 5.2 (−1.8 to 12.3) | 6.8 (−2.3 to 15.9) | 3.6 (−3.3 to 10.4) | 2.9 (1.7 to 4.1) | 0 | 6.9 (−6 to 19.8) |
| **Chronic cough, no TB** | 2.5 (0.7 to 4.4) | 4.1 (1.8 to 6.4) | 1.9 (−0.1 to 3.9) | 5.1 (2.2 to 8.1) | 4.7 (1.1 to 8.3) | 5.1 (0.2 to 10.0) | 0 | 5.1 (−0.5 to 10.8) | 3.8 (−3.5 to 11.2) | 10.5 (0.8 to 20.3) | 0 | 6.2 (−2.1 to 14.6) | 0 | 3.1 (1.9 to 4.4) | 6.3 (−5.6 to 18.1) |
| **Kidney disease** | 2.9 (0.9 to 4.9) | 2.2 (0.5 to 3.9) | 2.7 (0.5 to 4.9) | 3.3 (0.9 to 5.7) | 2.5 (−0.3 to 5.2) | 3.9 (−0.4 to 8.2) | 0 | 1.8 (−1.7 to 5.2) | 11.5 (−0.8 to 23.7) | 2.7 (−2.5 to 7.8) | 6.7 (−2.3 to 15.8) | 6.3 (−2.2 to 14.7) | 5.2 (−4.6 to 15.1) | 4.4 (−4 to 12.8) | 2.2 (1.1 to 3.3) |

N = 733 after multiple imputation of missing data. Top-left to bottom-right diagonal values represent the unconditional prevalence of the chronic condition.

**Table S2:** Prevalence of comorbidities among middle-aged and elderly women in peri-urban Tanzania (continued for women aged 50–59 years)

| Prevalence % [95% CI] | Index condition | | | | | | | | | | | | | | |
|---|---|---|---|---|---|---|---|---|---|---|---|---|---|---|---|
| **Comorbidity** | **Hypertension** | **Obese** | **Anemia** | **CES-D-10 score ≥10** | **Diabetes** | **Ischemic heart disease** | **Cognitive problems** | **HIV** | **High cholesterol** | **CAGE Score 2+** | **Tuberculosis** | **Stroke** | **Underweight** | **Chronic cough (no TB)** | **Kidney disease** |
| **Hypertension** | 54.4 (49.3 to 59.5) | 59.3 (51.8 to 66.9) | 46.8 (35.9 to 57.8) | 49.7 (40.8 to 58.6) | 55.8 (46.4 to 65.3) | 59 (46.7 to 71.4) | 67 (41.4 to 92.6) | 50.6 (31.4 to 69.9) | 67.8 (51.3 to 84.3) | 61.2 (42.3 to 80.1) | 41.7 (22 to 61.5) | 52.7 (30.2 to 75.2) | 50.9 (16.5 to 85.2) | 63.5 (34.9 to 92.0) | 58.2 (30.2 to 86.2) |
| **Anemia** | 48.6 (41.6 to 55.6) | 44.6 (39.5 to 49.7) | 36.1 (25 to 47.2) | 41.9 (33.1 to 50.6) | 51.5 (41.9 to 61.1) | 59.1 (46.7 to 71.4) | 67.6 (42 to 93.1) | 34.2 (16 to 52.4) | 66.8 (49.9 to 83.6) | 50.0 (30.7 to 69.3) | 21.5 (4.9 to 38.1) | 36.9 (15.1 to 58.7) | 0 | 27.2 (0.9 to 53.6) | 50 (21.6 to 78.4) |
| **Obese** | 25.8 (17.6 to 34.0) | 24.3 (15.2 to 33.4) | 30 (23.3 to 36.6) | 39.5 (28.1 to 50.9) | 27.5 (17.6 to 37.5) | 44.3 (28.1 to 60.4) | 50.2 (16.8 to 83.6) | 59.1 (35.2 to 83.1) | 26.2 (6.5 to 45.8) | 24.4 (3 to 45.8) | 52.0 (24.9 to 79.2) | 62.9 (35.5 to 90.4) | 42.4 (1.6 to 83.2) | 25.6 (−8.4 to 59.6) | 0 |
| **Signs of depression** | 30 (23.7 to 36.4) | 30.9 (23.8 to 38) | 43.4 (32.3 to 54.6) | 32.9 (28.1 to 37.7) | 29.4 (20.5 to 38.2) | 54.1 (41.6 to 66.7) | 49.9 (23.7 to 76.2) | 37.9 (19.3 to 56.5) | 19.4 (5.4 to 33.4) | 26.9 (9.8 to 44.0) | 59.9 (40.7 to 79.2) | 47.4 (24.9 to 69.9) | 38.3 (4.9 to 71.7) | 18.3 (−4.7 to 41.2) | 25.0 (0.4 to 49.6) |
| **Diabetes** | 39.7 (30.5 to 49.0) | 44.7 (34.3 to 55.1) | 35.6 (23.4 to 47.8) | 34.5 (23.7 to 45.4) | 38.7 (31.4 to 46) | 42.4 (26 to 58.7) | 42.8 (8.4 to 77.2) | 40.7 (16.4 to 64.9) | 49.6 (28.7 to 70.5) | 47.9 (24.3 to 71.5) | 28.0 (1.9 to 54.2) | 64.8 (37.1 to 92.5) | 0 | 60.8 (20.8 to 100) | 38.4 (3.7 to 73.0) |
| **Ischemic heart disease** | 17.6 (12.3 to 22.8) | 21.5 (15.2 to 27.7) | 24 (14.6 to 33.3) | 26.6 (18.8 to 34.4) | 17.7 (10.4 to 25.1) | 16.2 (12.5 to 19.9) | 28.5 (4.8 to 52.3) | 19 (3.9 to 34) | 29.1 (13 to 45.1) | 26.9 (9.8 to 44.0) | 44.0 (24.5 to 63.5) | 42.1 (19.8 to 64.4) | 0 | 45.3 (15.8 to 74.8) | 8.3 (−7.3 to 24.0) |
| **Cognitive impairment symptoms** | 4.6 (1.7 to 7.5) | 5.6 (2.1 to 9.2) | 6.2 (1.1 to 11.4) | 5.6 (1.6 to 9.7) | 4.1 (0.3 to 7.9) | 6.5 (0.3 to 12.8) | 3.7 (1.8 to 5.6) | 3.8 (−3.5 to 11.1) | 6.4 (−2.2 to 15.1) | 0 | 8.0 (−2.7 to 18.6) | 5.3 (−4.8 to 15.3) | 0 | 0 | 0 |
| **HIV** | 6.5 (3.1 to 9.9) | 5.3 (1.9 to 8.8) | 13.8 (6.5 to 21.2) | 8 (3.2 to 12.8) | 7.3 (2.4 to 12.2) | 8.2 (1.3 to 15.1) | 7.1 (−6.4 to 20.7) | 7 (4.4 to 9.6) | 9.7 (−0.8 to 20.1) | 16.8 (1.9 to 31.7) | 23.9 (7.1 to 40.6) | 10.5 (−3.3 to 24.3) | 0 | 0 | 8.3 (−7.3 to 24) |
| **High cholesterol** | 10.2 (6.1 to 14.4) | 12.3 (7.3 to 17.3) | 7.2 (1.3 to 13.1) | 4.8 (1.1 to 8.6) | 10.6 (5.1 to 16.0) | 14.8 (5.8 to 23.7) | 14.3 (−4.1 to 32.6) | 11.4 (−0.8 to 23.5) | 8.2 (5.4 to 11.0) | 7.7 (−2.6 to 18.0) | 12.0 (−0.8 to 24.9) | 15.9 (−0.6 to 32.4) | 11.8 (−10.1 to 33.8) | 9.0 (−7.9 to 26.0) | 16.6 (−4.5 to 37.8) |
| **Signs of alcohol problems** | 7.7 (4.1 to 11.4) | 7.7 (3.7 to 11.8) | 5.6 (0.4 to 10.7) | 5.6 (1.6 to 9.7) | 8.5 (3.3 to 13.7) | 11.4 (3.4 to 19.4) | 0 | 16.5 (2.0 to 31.0) | 6.4 (−2.2 to 15.1) | 6.9 (4.3 to 9.4) | 8 (−2.7 to 18.6) | 0 | 0 | 9.0 (−7.9 to 26.0) | 0 |
| **Tuberculosis** | 5.1 (2 to 8.2) | 3.2 (0.5 to 5.9) | 11.6 (4.3 to 18.9) | 12.1 (6.4 to 17.9) | 4.8 (0.1 to 9.4) | 18.1 (8.4 to 27.8) | 14.3 (−4.1 to 32.6) | 22.7 (6.7 to 38.8) | 9.7 (−0.8 to 20.2) | 7.7 (−2.6 to 18.0) | 6.7 (4.1 to 9.2) | 15.8 (−0.7 to 32.2) | 0 | 0 | 0 |
| **Stroke** | 4.9 (1.9 to 7.8) | 4.2 (1.1 to 7.2) | 10.6 (4.1 to 17.1) | 7.3 (2.7 to 11.8) | 8.4 (3.4 to 13.5) | 13.1 (4.6 to 21.6) | 7.1 (−6.4 to 20.7) | 7.6 (−2.6 to 17.7) | 9.7 (−0.8 to 20.2) | 0 | 11.9 (−0.8 to 24.6) | 5.0 (2.8 to 7.3) | 0 | 27.2 (0.9 to 53.6) | 16.6 (−4.5 to 37.8) |
| **Underweight** | 2.1 (0.1 to 4.2) | 0 | 3.2 (−0.6 to 7) | 2.6 (−0.3 to 5.6) | 0 | 0 | 0 | 0 | 3.2 (−3.0 to 9.5) | 0 | 0 | 0 | 2.3 (0.7 to 3.8) | 0 | 0 |
| **Chronic cough, no TB** | 3.4 (0.9 to 5.9) | 1.8 (−0.2 to 3.8) | 2.5 (−1.2 to 6.2) | 1.6 (−0.6 to 3.9) | 4.6 (0.6 to 8.5) | 8.2 (1.3 to 15.1) | 0 | 0 | 3.2 (−3.0 to 9.4) | 3.8 (−3.6 to 11.3) | 0 | 15.8 (−0.7 to 32.3) | 0 | 2.9 (1.2 to 4.6) | 8.3 (−7.3 to 24) |
| **Kidney disease** | 3.4 (0.9 to 5.9) | 3.6 (0.8 to 6.4) | 0 | 2.4 (−0.3 to 5.1) | 3.2 (−0.2 to 6.5) | 1.6 (−1.6 to 4.8) | 0 | 3.8 (−3.5 to 11.1) | 6.4 (−2.2 to 15.1) | 0 | 0 | 10.5 (−3.3 to 24.3) | 0 | 9.0 (−7.9 to 26.0) | 3.2 (1.4 to 5.0) |

N = 378 after multiple imputation of missing data. Top-left to bottom-right diagonal values represent the unconditional prevalence of the chronic condition.

**Table S2:** Prevalence of comorbidities among middle-aged and elderly women in peri-urban Tanzania (continued for women aged 60+ years)

| Prevalence % [95% CI] | Index condition | | | | | | | | | | | | | | |
|---|---|---|---|---|---|---|---|---|---|---|---|---|---|---|---|
| **Comorbidity** | **Hyper-tension** | **Obese** | **Anemia** | **CES-D-10 score ≥10** | **Diabetes** | **Ischemic heart disease** | **Cognitive problems** | **HIV** | **High chol-esterol** | **CAGE Score 2+** | **Tuber-culosis** | **Stroke** | **Under-weight** | **Chronic cough (no TB)** | **Kidney disease** |
| **Hypertension** | 71.2 (66.1 to 76.4) | 80.5 (72.5 to 88.5) | 67.1 (56.9 to 77.3) | 71.3 (62.9 to 79.7) | 70.9 (60.1 to 81.6) | 70.7 (59.7 to 81.8) | 68.2 (56.5 to 80) | 40.3 (9.8 to 70.8) | 78.3 (61.4 to 95.2) | 75.0 (50.4 to 99.6) | 44.6 (18.7 to 70.6) | 78.9 (60.6 to 97.3) | 58.8 (38.9 to 78.7) | 73.6 (53.7 to 93.5) | 78.9 (60.5 to 97.3) |
| **Anemia** | 37.2 (30.6 to 43.8) | 32.9 (27.5 to 38.3) | 27.9 (17.8 to 38) | 33.2 (24.3 to 42.2) | 38.1 (26.8 to 49.3) | 36.7 (24.7 to 48.7) | 16.1 (5.6 to 26.5) | 0 | 65 (45.4 to 84.6) | 27.6 (1.1 to 54.2) | 27.6 (4.4 to 50.8) | 25.9 (6.3 to 45.5) | 0 | 26.6 (6.4 to 46.8) | 46.8 (24.4 to 69.3) |
| **Obese** | 35 (25.9 to 44.2) | 31.4 (19.9 to 42.9) | 37.2 (29.4 to 45) | 39.1 (27.0 to 51.2) | 34.0 (21.8 to 46.2) | 45.1 (29.3 to 60.9) | 46.3 (30 to 62.6) | 48 (10.5 to 85.4) | 22.7 (0.4 to 45.0) | 46.3 (13.4 to 79.2) | 42.3 (9.4 to 75.3) | 48.8 (21.2 to 76.4) | 54.9 (28.6 to 81.2) | 25.8 (−2.2 to 53.9) | 38.2 (9.6 to 66.7) |
| **Signs of depression** | 38.1 (31.6 to 44.6) | 38.4 (28.7 to 48.1) | 40.0 (29.3 to 50.7) | 38.1 (32.6 to 43.5) | 36.4 (25.1 to 47.7) | 62.1 (50.4 to 73.9) | 43.9 (31.5 to 56.3) | 30.4 (1.7 to 59.1) | 22.0 (4.9 to 39) | 8.8 (−7.6 to 25.2) | 60.6 (35.8 to 85.3) | 40.6 (19.0 to 62.2) | 50.7 (30.7 to 70.8) | 22.7 (3.5 to 41.9) | 32.2 (11.1 to 53.3) |
| **Diabetes** | 33.8 (24.4 to 43.2) | 39.2 (26.8 to 51.6) | 31.0 (19.4 to 42.6) | 32.5 (20.6 to 44.4) | 33.9 (26.3 to 41.6) | 30.0 (15.8 to 44.3) | 38.2 (21.8 to 54.6) | 38.5 (1.7 to 75.3) | 45.5 (20.9 to 70.2) | 42.6 (8.9 to 76.3) | 0 | 48.2 (19.6 to 76.8) | 29.1 (5.9 to 52.4) | 55.1 (25 to 85.1) | 37.6 (8.1 to 67.1) |
| **Ischemic heart disease** | 21.1 (15.7 to 26.5) | 23.7 (15.3 to 32.1) | 25.8 (16.3 to 35.3) | 34.7 (26.0 to 43.3) | 18.8 (10.0 to 27.5) | 21.2 (16.7 to 25.8) | 24.6 (14.1 to 35.1) | 0 | 26.1 (8.1 to 44.0) | 41.4 (13.4 to 69.3) | 26.9 (4.4 to 49.5) | 15.4 (−0.6 to 31.4) | 23.1 (6.3 to 39.9) | 10.9 (−3.3 to 25) | 31.3 (10.5 to 52.2) |
| **Cognitive impair-ment symptoms** | 20.3 (14.9 to 25.7) | 10.3 (3.6 to 17.0) | 26.4 (16.8 to 36.1) | 24.4 (16.5 to 32.3) | 23.8 (13.9 to 33.7) | 24.5 (14.1 to 34.9) | 21.2 (16.6 to 25.7) | 0 | 0 | 17.2 (−4.4 to 38.9) | 13.7 (−3.9 to 31.3) | 24.1 (4.5 to 43.6) | 42.5 (22.8 to 62.1) | 43.8 (21.4 to 66.1) | 16.8 (−0.3 to 33.9) |
| **HIV** | 1.8 (0 to 3.6) | 0 | 4.2 (0 to 8.4) | 2.6 (−0.3 to 5.5) | 3.7 (−0.5 to 7.9) | 0 | 0 | 3.2 (1.3 to 5.2) | 0 | 0 | 0 | 0 | 3.9 (−3.6 to 11.3) | 0 | 0 |
| **High cholesterol** | 8.1 (4.5 to 11.8) | 14.6 (7.7 to 21.5) | 4.5 (−0.2 to 9.2) | 4.3 (0.6 to 8) | 10.0 (3.4 to 16.5) | 9.1 (2.1 to 16.1) | 0 | 0 | 7.4 (4.5 to 10.3) | 16.6 (−4.5 to 37.6) | 13.1 (−3.9 to 30.2) | 14.9 (−0.7 to 30.5) | 0 | 5.3 (−4.8 to 15.4) | 15.6 (−0.7 to 32) |
| **Signs of alcohol problems** | 4.1 (1.5 to 6.7) | 3.3 (−0.3 to 6.8) | 4.9 (0.5 to 9.2) | 0.9 (−0.9 to 2.7) | 4.9 (0.1 to 9.6) | 7.6 (1.2 to 13.9) | 3.2 (−1.2 to 7.5) | 0 | 8.7 (−2.9 to 20.2) | 3.9 (1.7 to 6) | 0 | 9.9 (−3.2 to 23) | 4.2 (−3.8 to 12.2) | 10.3 (−3.3 to 24.0) | 0 |
| **Tuberculosis** | 3.1 (0.7 to 5.4) | 4.1 (0.2 to 8.0) | 5.6 (0.4 to 10.8) | 7.8 (2.9 to 12.7) | 0 | 6.2 (0.3 to 12.1) | 3.2 (−1.2 to 7.5) | 0 | 8.7 (−2.9 to 20.2) | 0 | 4.9 (2.5 to 7.3) | 15.4 (−0.7 to 31.4) | 8.6 (−2.8 to 20) | 0 | 5.3 (−4.8 to 15.3) |
| **Stroke** | 7.2 (3.7 to 10.6) | 5.1 (0.8 to 9.5) | 8.5 (2.6 to 14.5) | 6.9 (2.3 to 11.6) | 9.2 (2.7 to 15.8) | 4.7 (−0.5 to 9.9) | 7.4 (0.8 to 14) | 0 | 13 (−0.8 to 26.8) | 16.6 (−4.5 to 37.8) | 20.4 (−0.1 to 40.9) | 6.5 (3.7 to 9.2) | 8.9 (−2.7 to 20.5) | 21.0 (2.6 to 39.4) | 16.0 (−0.6 to 32.5) |
| **Underweight** | 7.2 (3.5 to 10.8) | 0 | 12.8 (5.4 to 20.2) | 11.6 (5.4 to 17.8) | 7.4 (1.2 to 13.6) | 9.4 (2 to 16.9) | 17.4 (7.4 to 27.5) | 10.3 (−8.8 to 29.4) | 0 | 9.4 (−7.9 to 26.7) | 15.2 (−3.9 to 34.4) | 11.8 (−3.1 to 26.8) | 8.7 (5.4 to 12.0) | 8.3 (−5.7 to 22.4) | 11.4 (−3.3 to 26.2) |
| **Chronic cough, no TB** | 6.5 (3.2 to 9.8) | 5.1 (0.7 to 9.4) | 4.4 (−0.7 to 9.4) | 3.8 (0.2 to 7.4) | 10.2 (3.2 to 17.2) | 3.2 (−1.2 to 7.6) | 13 (4.6 to 21.4) | 0 | 4.5 (−4.1 to 13.1) | 16.7 (−4.5 to 37.9) | 0 | 20.3 (2.5 to 38) | 6.1 (−4.4 to 16.5) | 6.3 (3.5 to 9.0) | 10.8 (−3.3 to 25) |
| **Kidney disease** | 6.8 (3.5 to 10.2) | 8.8 (3.3 to 14.3) | 6.3 (1.0 to 11.7) | 5.2 (1.1 to 9.3) | 6.8 (0.8 to 12.9) | 9.1 (2.1 to 16.1) | 4.9 (−0.5 to 10.3) | 0 | 13 (−0.8 to 26.8) | 0 | 6.6 (−6 to 19.2) | 15.2 (−0.7 to 31) | 8.2 (−2.7 to 19.1) | 10.7 (−3.3 to 24.6) | 6.2 (3.5 to 8.9) |

N = 312 after multiple imputation of missing data. Top-left to bottom-right diagonal values represent the unconditional prevalence of the chronic condition.

**Table S3:** Prevalence and extent of multimorbidity among middle-aged and elderly women in peri-urban Tanzania, by age-group

| Number of chronic conditions or health areas affected | Age group | Prevalence of chronic conditions % (95% CI) | Prevalence of health areas affected % (95% CI) |
|---|---|---|---|
| 0 | 40–44 | 9.7 (6.4 to 12.9) | 9.7 (6.4 to 12.9) |
| | 45–49 | 6.6 (3.5 to 9.7) | 6.6 (3.5 to 9.7) |
| | 50–54 | 4.3 (1.3 to 7.3) | 4.3 (1.3 to 7.3) |
| | 55–59 | 6.7 (2.5 to 10.9) | 6.7 (2.5 to 10.9) |
| | 60–64 | 3.3 (0 to 6.7) | 3.3 (0 to 6.7) |
| | 65–69 | 2.3 (−1.4 to 6.1) | 2.3 (−1.4 to 6.1) |
| | 70+ | 0 | 0 |
| 1 | 40–44 | 26.2 (21.6 to 30.8) | 54 (49.1 to 58.9) |
| | 45–49 | 21.9 (16.7 to 27.2) | 56.8 (51.2 to 62.5) |
| | 50–54 | 19.1 (12.9 to 25.3) | 51.5 (44.9 to 58.2) |
| | 55–59 | 15.6 (9.4 to 21.9) | 55.7 (47.9 to 63.5) |
| | 60–64 | 15.5 (8.3 to 22.6) | 51.1 (42.3 to 59.8) |
| | 65–69 | 10.3 (2.1 to 18.5) | 47.1 (36.0 to 58.3) |
| | 70+ | 7.4 (1.6 to 13.3) | 27.6 (18.5 to 36.7) |
| 2 | 40–44 | 25.9 (21.2 to 30.6) | 31.7 (27.4 to 36.1) |
| | 45–49 | 32.6 (26.7 to 38.6) | 31.2 (26.1 to 36.4) |
| | 50–54 | 26.9 (20.3 to 33.5) | 38.0 (31.7 to 44.4) |
| | 55–59 | 29.1 (21.3 to 37.0) | 32.3 (25.2 to 39.5) |
| | 60–64 | 30.5 (20.9 to 40.1) | 42.5 (34.0 to 51.1) |
| | 65–69 | 29.4 (17.3 to 41.5) | 45.4 (34.2 to 56.5) |
| | 70+ | 17.5 (7.7 to 27.2) | 69.2 (59.8 to 78.6) |
| 3 | 40–44 | 20.8 (16.4 to 25.2) | 4.6 (2.6 to 6.5) |
| | 45–49 | 21.6 (16.5 to 26.7) | 5.3 (2.9 to 7.8) |
| | 50–54 | 24.7 (18.1 to 31.2) | 6.2 (3.0 to 9.3) |
| | 55–59 | 24.1 (16.5 to 31.7) | 5.3 (1.9 to 8.6) |
| | 60–64 | 23.1 (14.6 to 31.5) | 3.1 (0.1 to 6.0) |
| | 65–69 | 29.3 (17.1 to 41.4) | 5.2 (0 to 10.4) |
| | 70+ | 26.2 (15.0 to 37.5) | 3.2 (−0.4 to 6.8) |
| 4 | 40–44 | 11.8 (8.4 to 15.2) | |
| | 45–49 | 10.1 (6.2 to 14.0) | |
| | 50–54 | 13.8 (8.5 to 19.1) | |
| | 55–59 | 12.2 (6.3 to 18.0) | |
| | 60–64 | 14.2 (6.8 to 21.6) | |
| | 65–69 | 15.2 (5.5 to 24.9) | |
| | 70+ | 26.9 (16.3 to 37.6) | |
| 5+ | 40–44 | 5.6 (3.2 to 8.0) | |
| | 45–49 | 7.1 (4.1 to 10.1) | |
| | 50–54 | 11.3 (6.7 to 15.8) | |
| | 55–59 | 12.2 (6.9 to 17.5) | |
| | 60–64 | 13.4 (6.8 to 20.1) | |
| | 65-69 | 13.5 (5.1 to 21.9) | |
| | 70+ | 21.9 (12.3 to 31.5) | |

N = 1528 after multiple imputation of missing data.

**Table S3:** Prevalence and extent of multimorbidity among middle-aged and elderly women in peri-urban Tanzania, by age-group (continued)

| Number of chronic conditions or health areas affected | Age group | Prevalence of chronic conditions % (95% CI) | Prevalence of health areas affected % (95% CI) |
|---|---|---|---|
| 0–1 | 40–44 | 35.9 (31.0 to 40.7) | 63.7 (59.2 to 68.2) |
| | 45–49 | 28.5 (22.8 to 34.3) | 63.4 (58.2 to 68.7) |
| | 50–54 | 23.4 (16.9 to 29.8) | 55.8 (49.3 to 62.3) |
| | 55–59 | 22.3 (15.3 to 29.3) | 62.4 (55.0 to 69.8) |
| | 60–64 | 18.8 (11.2 to 26.4) | 54.4 (45.8 to 63.0) |
| | 65-69 | 12.6 (4.0 to 21.3) | 49.5 (38.4 to 60.5) |
| | 70+ | 7.4 (1.6 to 13.3) | 27.6 (18.5 to 36.7) |
| 2+ | 40–44 | 64.1 (59.3 to 69.0) | 36.3 (31.8 to 40.8) |
| | 45–49 | 71.5 (65.7 to 77.2) | 36.6 (31.3 to 41.8) |
| | 50–54 | 76.6 (70.2 to 83.1) | 44.2 (37.7 to 50.7) |
| | 55–59 | 77.7 (70.7 to 84.7) | 37.6 (30.2 to 45.0) |
| | 60–64 | 81.2 (73.6 to 88.8) | 45.6 (37.0 to 54.2) |
| | 65-69 | 87.4 (78.7 to 96.0) | 50.5 (39.5 to 61.6) |
| | 70+ | 92.6 (86.7 to 98.4) | 72.4 (63.3 to 81.5) |
| 0–2 | 40–44 | 61.7 (56.8 to 66.7) | |
| | 45–49 | 61.2 (55.2 to 67.1) | |
| | 50–54 | 50.3 (43.1 to 57.5) | |
| | 55–59 | 51.5 (43.1 to 59.8) | |
| | 60–64 | 49.3 (39.3 to 59.3) | |
| | 65-69 | 42.1 (29.2 to 54.9) | |
| | 70+ | 24.9 (14.5 to 35.3) | |
| 3+ | 40–44 | 38.3 (33.3 to 43.2) | |
| | 45–49 | 38.8 (32.9 to 44.8) | |
| | 50–54 | 49.7 (42.5 to 56.9) | |
| | 55–59 | 48.5 (40.2 to 56.9) | |
| | 60–64 | 50.7 (40.7 to 60.7) | |
| | 65-69 | 57.9 (45.1 to 70.8) | |
| | 70+ | 75.1 (64.7 to 85.5) | |
| 0–3 | 40–44 | 82.6 (78.7 to 86.4) | |
| | 45–49 | 82.8 (78.2 to 87.4) | |
| | 50–54 | 74.9 (68.6 to 81.2) | |
| | 55–59 | 75.6 (68.3 to 82.9) | |
| | 60–64 | 72.4 (63.7 to 81.0) | |
| | 65-69 | 71.3 (60.0 to 82.6) | |
| | 70+ | 51.2 (39.3 to 63.0) | |
| 4+ | 40–44 | 17.4 (13.6 to 21.3) | |
| | 45–49 | 17.2 (12.6 to 21.8) | |
| | 50–54 | 25.1 (18.8 to 31.4) | |
| | 55–59 | 24.4 (17.1 to 31.7) | |
| | 60–64 | 27.6 (19.0 to 36.3) | |
| | 65-69 | 28.7 (17.4 to 40.0) | |
| | 70+ | 48.8 (37.0 to 60.7) | |

N = 1528 after multiple imputation of missing data.

**Figure S1:** Comorbidity patterns among middle-aged and elderly women in peri-urban Tanzania, by age group

(a) 40–49 years

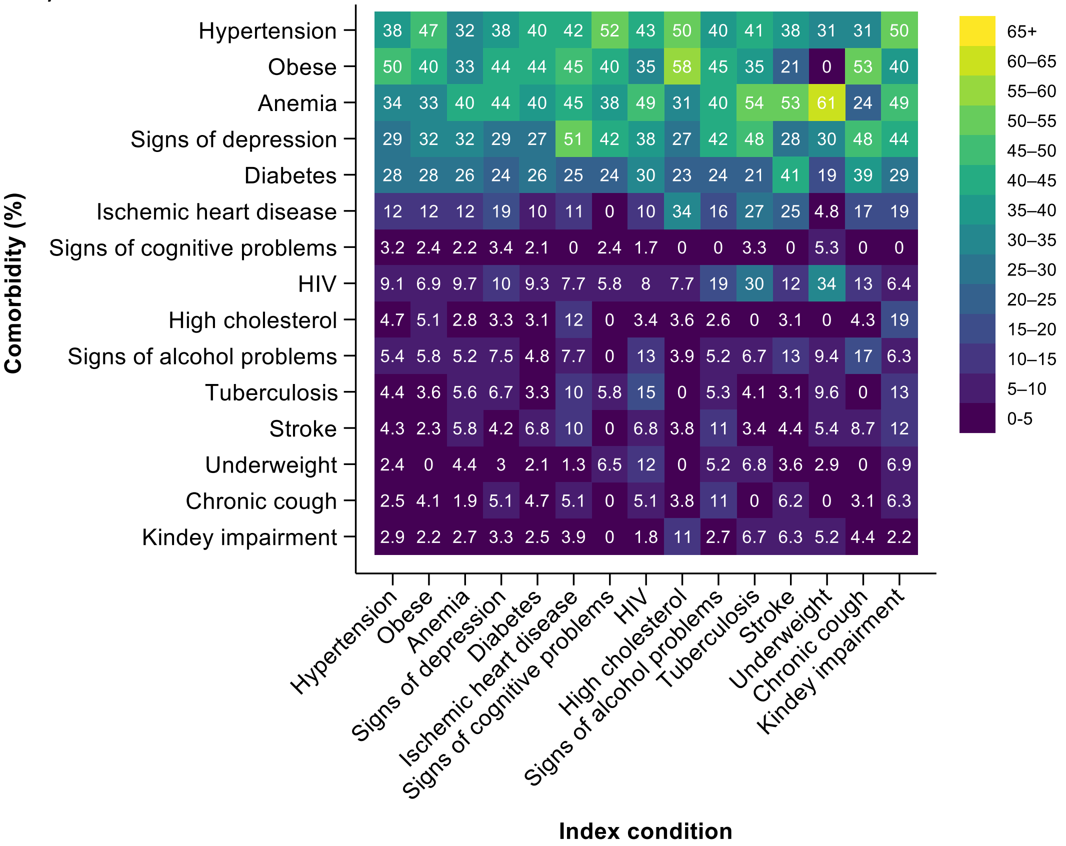


(b) 50–59 years

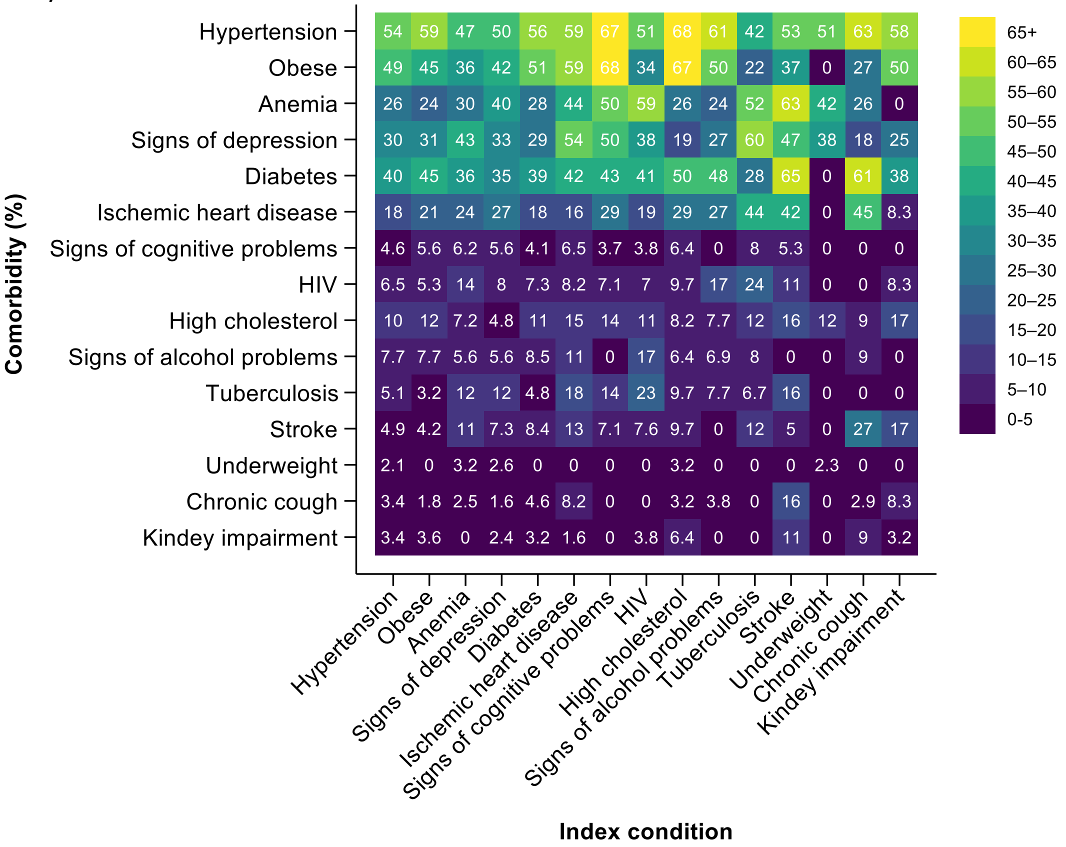

(d) 60+ years

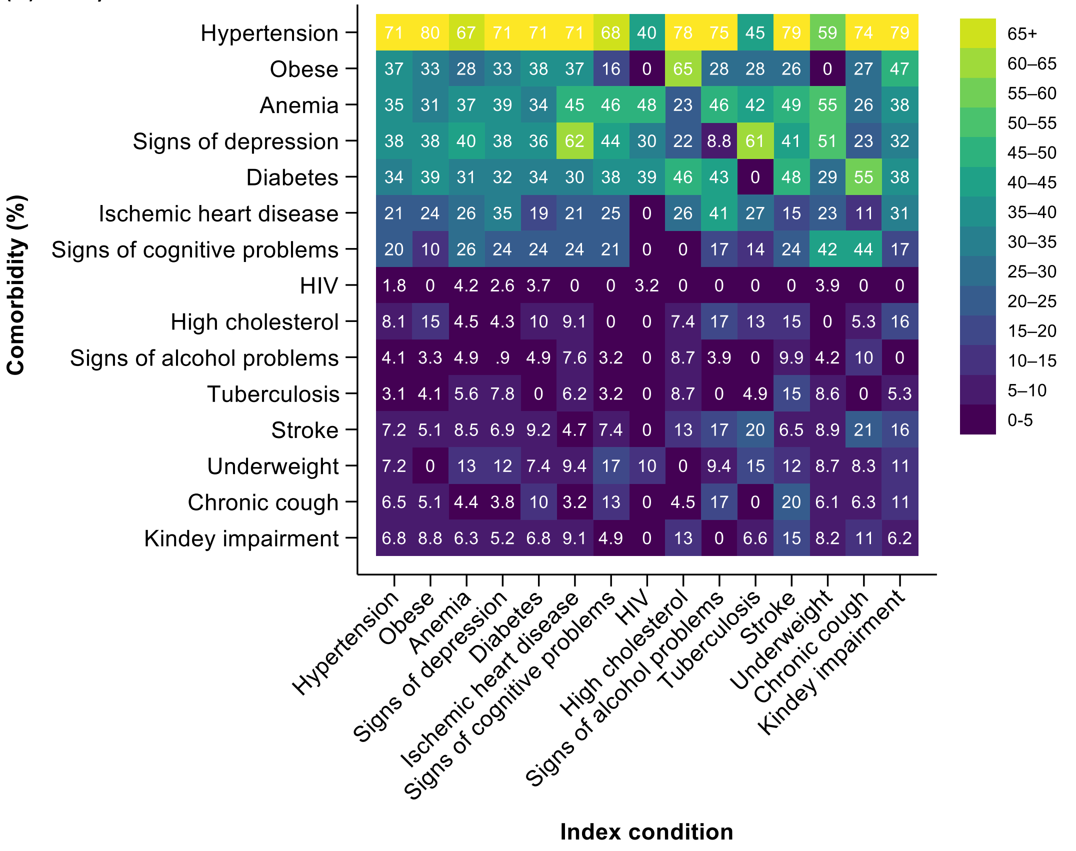


N = 733, 378 and 312, respectively, after multiple imputation of missing data. Off-diagonal values represent the conditional prevalence of a comorbidity given the presence of the index condition. Top-left to bottom-right diagonal values represent the unconditional prevalence of the chronic condition. Data with 95% confidence intervals are provided in **supplementary Table S2**.
